# The endoplasmic reticulum membrane complex promotes proteostasis of GABA_A_ receptors

**DOI:** 10.1016/j.isci.2022.104754

**Published:** 2022-07-13

**Authors:** Angela L. Whittsette, Ya-Juan Wang, Ting-Wei Mu

**Affiliations:** 1 Department of Physiology and Biophysics, Case Western Reserve University School of Medicine, 10900 Euclid Avenue, Cleveland, OH 44106, USA

**Keywords:** Biological sciences, Molecular biology, Neuroscience, Molecular neuroscience, Cell biology

## Abstract

The endoplasmic reticulum membrane complex (EMC) plays a critical role in the biogenesis of tail-anchored proteins and a subset of multi-pass membrane proteins in the endoplasmic reticulum (ER). However, because of nearly exclusive expression of neurotransmitter-gated ion channels in the central nervous system (CNS), the role of the EMC in their biogenesis is not well understood. In this study, we demonstrated that the EMC positively regulates the surface trafficking and thus function of endogenous γ-aminobutyric acid type A (GABA_A_) receptors, the primary inhibitory ion channels in the mammalian brain. Moreover, among ten EMC subunits, EMC3 and EMC6 have the most prominent effect, and overexpression of EMC3 or EMC6 is sufficient to restore the function of epilepsy-associated GABA_A_ receptor variants. In addition, EMC3 and EMC6 demonstrate endogenous interactions with major neuroreceptors, which depends on their transmembrane domains, suggesting a general role of the EMC in the biogenesis of neuroreceptors.

## Highlights

• EMC3 and EMC6 positively regulate the function of endogenous GABA_A_ receptors
• The EMC interacts with major endogenous neuroreceptors
• The EMC transmembrane domains contribute to the interaction with GABA_A_ receptors
• Overexpressing the EMC restores the function of GABA_A_ receptor variants

## Graphical abstract

**Figure:**
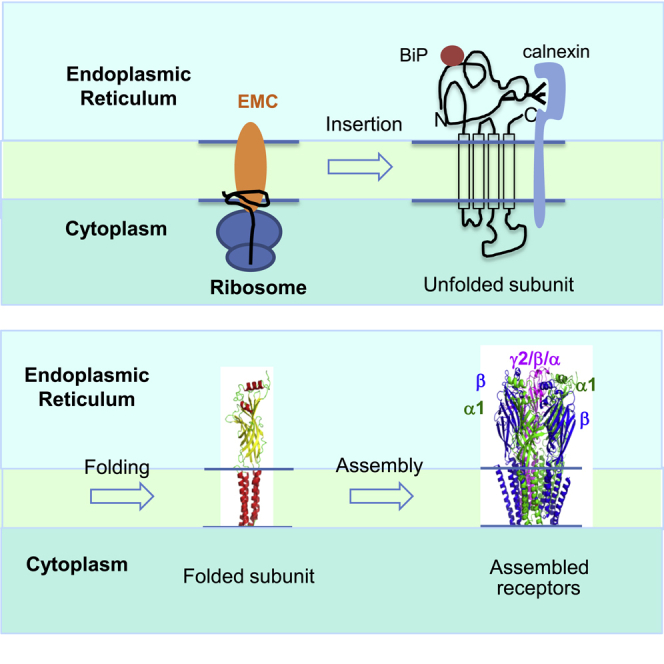

## Introduction

**Figure 1:**
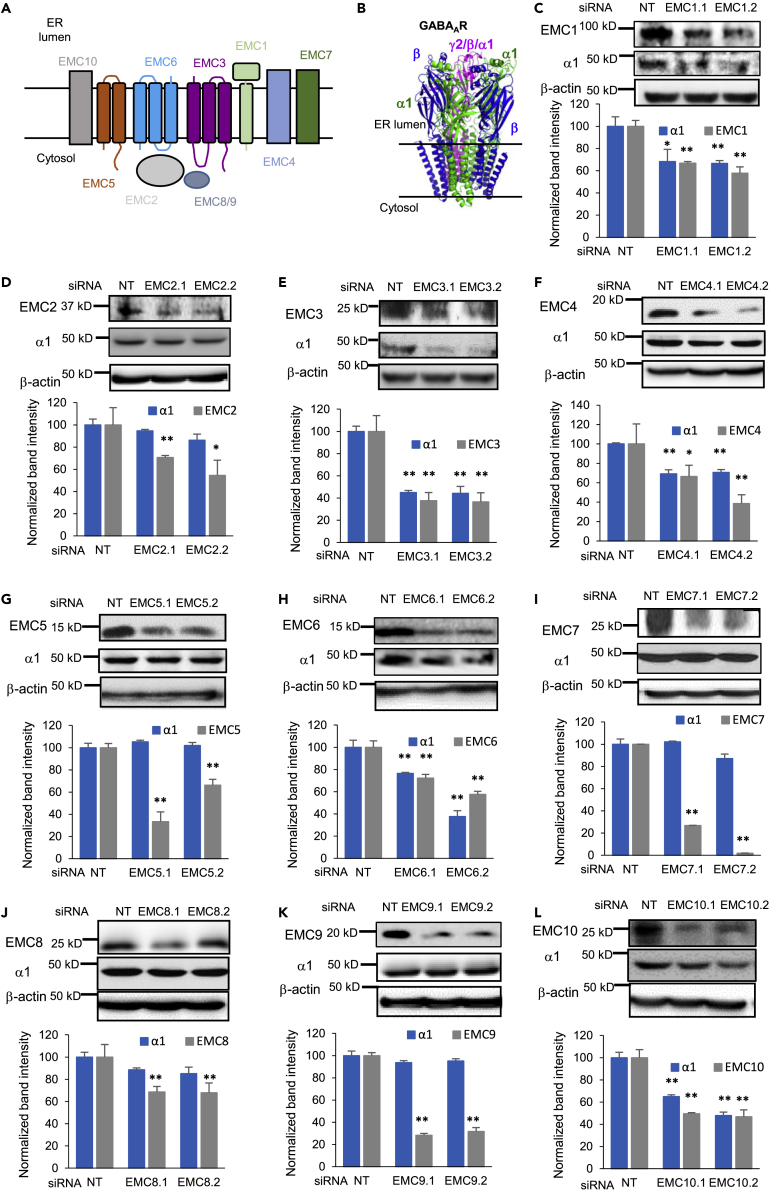
Effect of depleting individual EMC subunits on endogenous GABA_A_ receptor α1 subunit protein levels (A) Schematics of ten EMC subunits. (B) Cartoon Representation of heteropentameric GABA_A_ receptors. The most common subtype in the mammalian CNS consists of α1, β2/β3, and γ2 subunits. (C to L) Endogenous total GABA_A_ receptor α1 subunits protein level change on knocking down individual EMC subunits. Mouse hypothalamus GT1-7 neuronal cells were transfected with siRNAs against EMC1 to EMC10, respectively. Two distinct siRNAs targeting each of the ten EMC subunits, designated as EMCn.1 and EMCn.2 (n = 1 to 10), were used to minimize the potential off-target effects. Forty-eight hours after transfection, proteins were extracted and analyzed by western blotting. β-actin was used as the loading control. Normalized band intensity was shown below the images (n = 3). Each data point is presented as mean ± SEM ∗, p< 0.05; ∗∗, p< 0.01. NT: Non-targeting scrambled siRNA.

Acquiring the correct transmembrane topology is essential for the function of membrane proteins, which consist of about 30% of the eukaryotic proteome. The endoplasmic reticulum membrane complex (EMC) plays a critical role in the insertion of membrane proteins into the lipid bilayer of the endoplasmic reticulum (ER) (Chitwood et al., 2018; Guna et al., 2018; Shurtleff et al., 2018). The EMC is ubiquitously expressed and highly conserved (Volkmar and Christianson, 2020; Wideman, 2015). Ten subunits (EMC1-10) have been identified in the EMC in mammals: EMC1, EMC3-7, and EMC10 are membrane-spanning, whereas EMC2, EMC8, and EMC9 are soluble (EMC8 and EMC9 are structurally redundant and mutually exclusive) (Figure 1A) (O'Donnell et al., 2020). Recent cryo-electron microscope (cryo-EM) structures showed the overall similar organization of the human EMC (Pleiner et al., 2020) and yeast EMC (Bai et al., 2020): both have a large ER luminal region, 12 transmembrane helices, and a smaller cytosolic region. Research has shown that EMC1-3, 5, and 6 are the core subunits because their depletion leads to co-translational degradation of other subunits, malfunction in the assembly of the full mature EMC, and loss of EMC’s overall activity in U-2 OS cells (Volkmar et al., 2019).

A growing number of EMC-dependent client membrane proteins continue to be reported (Volkmar and Christianson, 2020). The EMC was first described in yeast in a genetic screen for protein folding factors (Jonikas et al., 2009). Later, the EMC was identified in the screen of the interactome for the mammalian ER-associated degradation (ERAD) network (Christianson et al., 2011). Recently, the EMC’s important role has been recognized in inserting tail-anchored membrane proteins post-translationally and a subset of multi-pass membrane proteins co-translationally. The EMC acts as an insertase for tail-anchored proteins with moderately hydrophobic transmembrane domains (Guna et al., 2018). Subsequently, the EMC was reported to facilitate the insertion of the first transmembrane domain of certain G-protein coupled receptors (GPCRs), such as β-adrenergic receptors (Chitwood et al., 2018). Furthermore, quantitative proteomics analysis comprehensively identified EMC-dependent membrane protein clients, showing that the EMC enables the biogenesis of membrane proteins with destabilizing features, such as polar and charged residues-containing transmembrane domains (Shurtleff et al., 2018; Tian et al., 2019). The idea that the EMC could act as a more general factor to facilitate the biogenesis of membrane proteins should be further investigated because the EMC regulates the function of a variety of membrane proteins, including cystic fibrosis transmembrane conductance regulator (CFTR) in yeast, acetylcholine receptors in *C. elegans*, and rhodopsins and transient receptor potential (TRP) channels in *Drosophila* (Louie et al., 2012; Richard et al., 2013; Satoh et al., 2015).

We focus on proteostasis maintenance of neurotransmitter-gated ion channels (Fu et al., 2016). Because of the nearly exclusive expression of such membrane proteins in the CNS, previous screenings in yeast and mammalian cells did not identify the potential involvement of the EMC in their biogenesis (Shurtleff et al., 2018; Tian et al., 2019). Interestingly, EMC6 was shown to positively regulate the expression of acetylcholine receptors and the response to GABA in *C. elegans* (Richard et al., 2013). Therefore, here we evaluated the role of the EMC in the biogenesis of GABA_A_ receptors, the primary inhibitory ion channels in the mammalian CNS (Akerman and Cline, 2007). Functional GABA_A_ receptors are assembled as a pentamer in the ER from eight subunit classes: α1-6, β1-3, γ1-3, δ, ε, θ, π, and ρ1-3 (Sequeira et al., 2019). The most common subtype in the human brain contains two α1 subunits, two β2/β3 subunits, and one γ2 subunit (Figure 1B) (Laverty et al., 2019; Zhu et al., 2018). Individual subunits must fold into their native structures in the ER and assemble with other subunits correctly on the ER membrane to form a heteropentamer (Barnes, 2001; Jacob et al., 2008; Martenson et al., 2017). Only properly assembled receptors exit the ER, traffic through the Golgi for complex glycosylation, and reach the plasma membrane for their proper function (Lorenz-Guertin and Jacob, 2018). The proteostasis network, which contains chaperones (such as BiP and calnexin), ERAD factors (such as Hrd1, Sel1L, and VCP), and trafficking factors (such as LMAN1), orchestrates the folding, assembly, degradation, and trafficking of GABA_A_ receptors, which is essential for their function (Di et al., 2013; Di et al., 2016; Fu et al., 2019; Wang et al., 2022b; Wang et al., 2013). Loss of function of GABA_A_ receptors causes a variety of neurological diseases, including epilepsy and intellectual disability (Hernandez and Macdonald, 2019). Furthermore, numerous clinical variants in a single subunit cause subunit protein misfolding and/or disrupt assembly of the pentameric complex in the ER (Fu et al., 2022). Such mutant subunits are retained in the ER and subjected to excessive ERAD, being dislocated into the cytosol and degraded by the proteasome. This leads to decreased cell surface localization of the receptor complex and imbalanced neural circuits. Because the proteostasis network orchestrates the biogenesis of GABA_A_ receptors, regulating the expression or function of their proteostasis network components, such as GABA_A_ receptor-interacting chaperones and ERAD factors, can fine-tune the functional surface expression of GABA_A_ receptors. Specifically, activating the pro-folding chaperones or inhibiting the ERAD factors genetically or pharmacologically has the potential to restore the surface trafficking and thus function of pathogenic GABA_A_ receptors, providing a therapeutic strategy to ameliorate related neurological diseases (Wang et al., 2022a; Wang et al., 2014). For example, overexpression of BiP, a pro-folding Hsp70 family chaperone in the ER, is sufficient to promote the anterograde trafficking and surface expression of pathogenic GABA_A_ receptors containing the A322D mutation in the α1 subunit (Di et al., 2013). Consistently, increasing the BiP protein levels pharmacologically through the application of a small molecule BiP activator called BIX leads to enhanced functional surface expression of this variant (Fu et al., 2018).

Here we aim to understand the role of each individual subunit of the EMC on the proteostasis maintenance of GABA_A_ receptors. We found that EMC3 and EMC6 have the most prominent effect on increasing the protein levels of endogenous GABA_A_ receptors. Furthermore, the interactions between the EMC and GABA_A_ receptors are dependent on the transmembrane domains. Overexpressing the EMC is sufficient to restore the function of certain disease-associated variants of GABA_A_ receptors.

## Results

### Knocking down EMC3 and EMC6 reduces the protein levels and function of endogenous GABA_A_ receptors

We evaluated the effect of each of the ten individual EMC subunits on endogenous GABA_A_ receptor protein levels. As a starting point, we used a mouse GT1-7 hypothalamic neuronal cell line because it is one of the very few neuronal cells that express endogenous, functional GABA_A_ receptors (with α1 and β3 subunits) (Hales et al., 1992). In addition, GT1-7 is a mature mouse hypothalamic gonadotropin releasing hormone cell line that responds to membrane depolarization (Hales et al., 1994), which is a defining characteristic of neurotransmitter-gated ion channels. To investigate whether there are key subunits of the EMC that play critical roles in the biogenesis of GABA_A_ receptors, we conducted siRNA screening by using two distinct siRNAs targeting each of the ten EMC subunits to minimize the potential off-target effects. GT1-7 cells have high transfection efficiency, which also made them suitable for the siRNA screening experiments (Fu et al., 2019). To further increase knockdown efficiency, we performed two siRNA transfections at 24 and 48 h before harvesting the proteins for SDS-poly acrylamide gel electrophoresis (PAGE) and western blot analysis (Figures 1C–1L).

Of the five core subunits of the EMC (EMC1-3, 5, and 6) that are critical for its assembly and activity, EMC1, EMC3 and EMC6 positively regulated α1 protein levels (Figures 1C, 1E and 1H), whereas the knockdown of EMC2 or EMC5 did not influence the α1 protein levels significantly (Figures 1D and 1G), suggesting that the formation of the mature EMC might not be a prerequisite for the regulation of GABA_A_ receptor protein levels. The most substantial reduction of the total α1 protein levels was observed with the depletion of EMC3 (p*<*0.01, Figure 1E) and EMC6 (p*<*0.01, Figure 1H). This is noted in the figures as decreased EMC3 band intensity for EMC3.1 and EMC3.2 siRNAs-treated samples, compared to the non-targeting (NT) control, to 38 and 37% respectively (Figure 1E). Correspondingly, when α1 was detected, two decreased band intensities were observed as well, to 41 and 45% respectively (Figure 1E). We also noted significant effects on EMC6 knockdown (p< 0.01, Figure 1H) as well. This is noted as decreased EMC6 band intensity for EMC6.1 and EMC6.2 siRNAs-treated samples, compared to NT, to 70 and 58% respectively. Correspondingly, when α1 was detected, two decreased band intensities were observed as well, to 75 and 35% respectively (Figure 1H). Therefore, our results suggested that individual EMC subunit, such as EMC3 and EMC6, is sufficient to positively regulate GABA_A_ receptor protein levels (also see below). EMC3 is the only EMC subunit that exhibits homology to Oxa1 family proteins, which are known membrane protein insertases (Volkmar and Christianson, 2020; Wideman, 2015), and EMC6 plays a critical role in regulating the biogenesis of acetylcholine receptors in *C. elegans* (Richard et al., 2013).

Among the five peripheral subunits of the EMC (EMC4, 7-10) that are not essential for the complex’s stability or assembly, EMC4 and EMC10 positively regulated α1 protein levels (Figures 1F and 1L), whereas the knockdown of EMC7-9 did not influence the α1 protein levels significantly (Figures 1I, 1J and 1K), again suggesting a subunit-specific contribution of the EMC. It is worth noting that all cytosolic, soluble EMC subunits (EMC2, 8-9) did not influence α1 protein levels significantly, suggesting that the transmembrane domains in other membrane-spanning EMC subunits could play a critical role in regulating GABA_A_ receptor biogenesis in the ER membrane (also see below).

**Figure 2:**
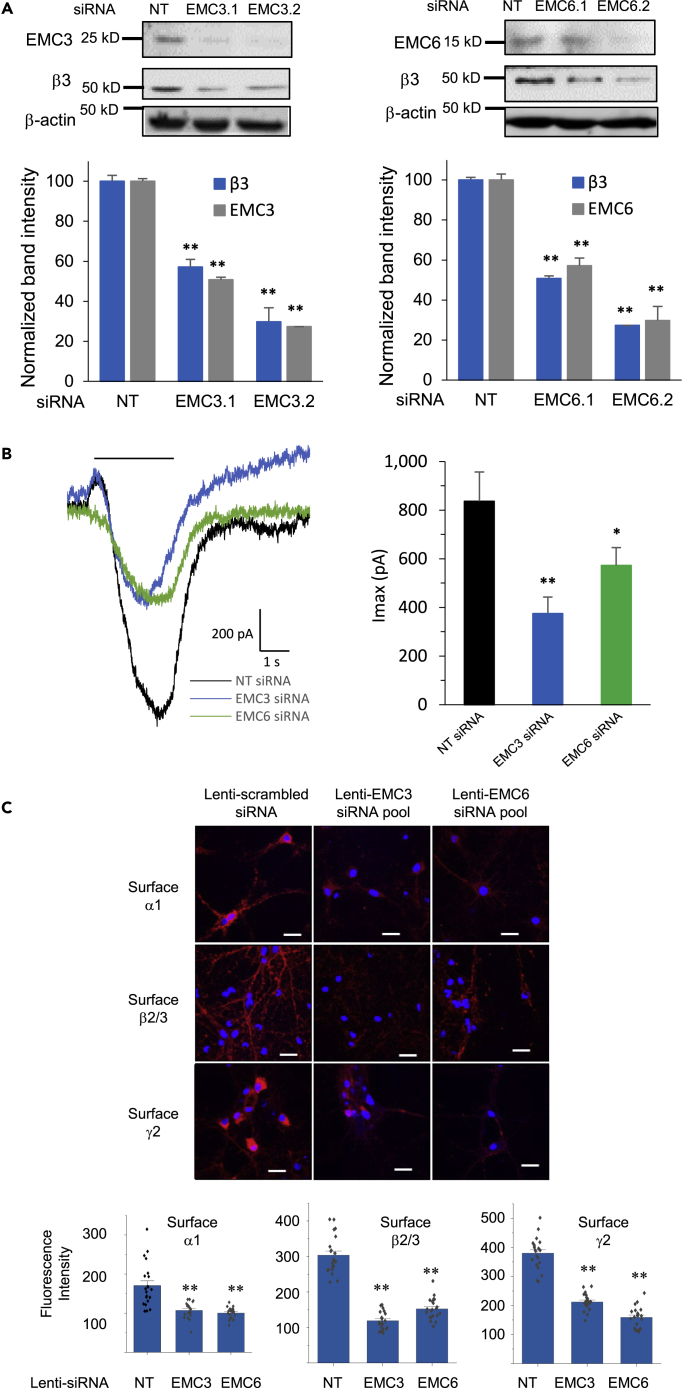
Effect of EMC3 and EMC6 on the protein levels and whole-cell patch-clamping currents of endogenous GABA_A_receptors (A) Mouse GT1-7 neurons were incubated with siRNA against EMC3 or EMC6 for 48 h. Proteins were extracted and analyzed by western blotting; normalized band intensity was shown below the images (n = 3), with β-actin as the loading control. (B) Mouse GT1-7 neurons were incubated with siRNA against EMC3 or EMC6 for 48 h. Whole-cell patch-clamping was performed on the cells with the IonFlux Mercury 16 ensemble plates at a holding potential of −60 mV. GABA (1 mM) was applied for 4 s, as indicated by the horizontal bar above the currents. The peak currents (Imax) were acquired and analyzed by Fluxion Data Analyzer (n = 6 - 10). NT: Non-targeting scrambled siRNA; pA, picoampere. (C) Confocal microscopy imaging of primary rat cortical neurons demonstrated reduced surface expression of GABA_A_ receptors after siRNA treatment of EMC3 and EMC6 through lentivirus transduction. Lentiviruses were generated from transiently transfected HEK293T cells with the following plasmids and collected after 60 h from the media passing through 0.45 μm filter: EMC3- or EMC6-set of four siRNA lentivectors, packaging and envelop plasmids. At day-in-vitro (DIV) 6 of the primary rat cortical neurons, lentivirus transduction was carried out at a multiplicity-of-infection (MOI) of 10. At DIV 12, neurons were stained for cell surface GABA_A_ receptor α1 subunits (top row), β2/β3 subunits (middle row), and γ2 subunits (bottom row), colored in red. DAPI staining for the nucleus was colored in blue. Scale bar = 20 μm. Quantification of the fluorescence intensity by using ImageJ was shown on the bottom after background correction from 20–30 neurons. Each data point is presented as mean ± SEM ∗, p< 0.05; ∗∗, p< 0.01.

In addition, we performed experiments to detect endogenous β3 protein levels from EMC3 and EMC6 knockdown samples in GT1-7 cells to further determine their effects on other major subunits of GABA_A_ receptors (Figure 2A). Knocking down EMC3 by using two distinct siRNAs decreased the EMC3 protein levels to 50 and 25% compared to the non-targeting control (Figure 2A, left panel) and reduced the endogenous β3 protein levels to 55 and 30%, respectively (Figure 2A, left panel). Moreover, knocking down EMC6 by using two distinct siRNAs decreased the EMC6 protein levels to 55 and 30% compared to the non-targeting control (Figure 2A, right panel) and reduced the endogenous β3 protein levels to 50 and 25%, respectively (Figure 2A, right panel). The results from Figures 1 and 2A clearly indicated that EMC3 and EMC6 positively regulate the protein levels of the endogenous α1 and β3 subunits of GABA_A_ receptors.

To understand their effects on the function of endogenous GABA_A_ receptors, we carried out whole-cell patch-clamping electrophysiological recording using GT1-7 neurons after siRNA treatment of EMC3 and EMC6. The peak GABA-induced current in the non-targeting siRNA-treated GT1-7 neurons is 837 pA; consistently, knocking down EMC3 or EMC6 decreased the peak current (Imax) to 375 and 573 pA, corresponding to 55 and 32% reduction respectively (Figure 2B). These results unambiguously demonstrated EMC3 and EMC6 as key EMC subunits to positively regulate protein expression and function of endogenous GABA_A_ receptors.

### EMC3 and EMC6 promote anterograde trafficking of GABA_A_ receptors

Because GABA_A_ receptors need to traffic to the plasma membrane for their function, we further tested the hypothesis about the necessity of the EMC with respect to surface trafficking of endogenous GABA_A_ receptors using immunofluorescence staining. We used primary rat cortical neurons because all of the major subunits (α1, β2, β3, and γ2 subunits) of functional GABA_A_ receptors are abundantly expressed in the plasma membrane in the cortex (Sequeira et al., 2019). EMC3 and EMC6 depletion was achieved through the lentivirus transduction of four siRNA pools (Ding and Kilpatrick, 2013). At day-in-vitro (DIV) 6 of the neurons, lentivirus transduction was carried out at a MOI of 10, that is, the ratio of lentivirus count to neuron count in each well. Immunofluorescence staining was performed at DIV 12 (Glynn and McAllister, 2006). The application of an anti-GABA_A_ receptor subunit antibody that recognizes the extracellular epitope without a prior membrane permeabilization step using detergent enabled us to label the cell surface proteins of α1, β2/3 and γ2 subunits. Application of the Alexa Fluor Dye-conjugated secondary antibody enabled the imaging of the surface subunits using a confocal laser-scanning microscope. Clearly, depletion of EMC3 and EMC6 reduced the surface expression of α1 subunits by 37 and 41%, β2/3 subunits by 61 and 50%, and γ2 subunits by 44 and 58% in cortical neurons (Figure 2C), indicating that EMC3 and EMC6 play a critical role in positively regulating the surface trafficking of all major subunits of endogenous GABA_A_ receptors.

**Figure 3:**
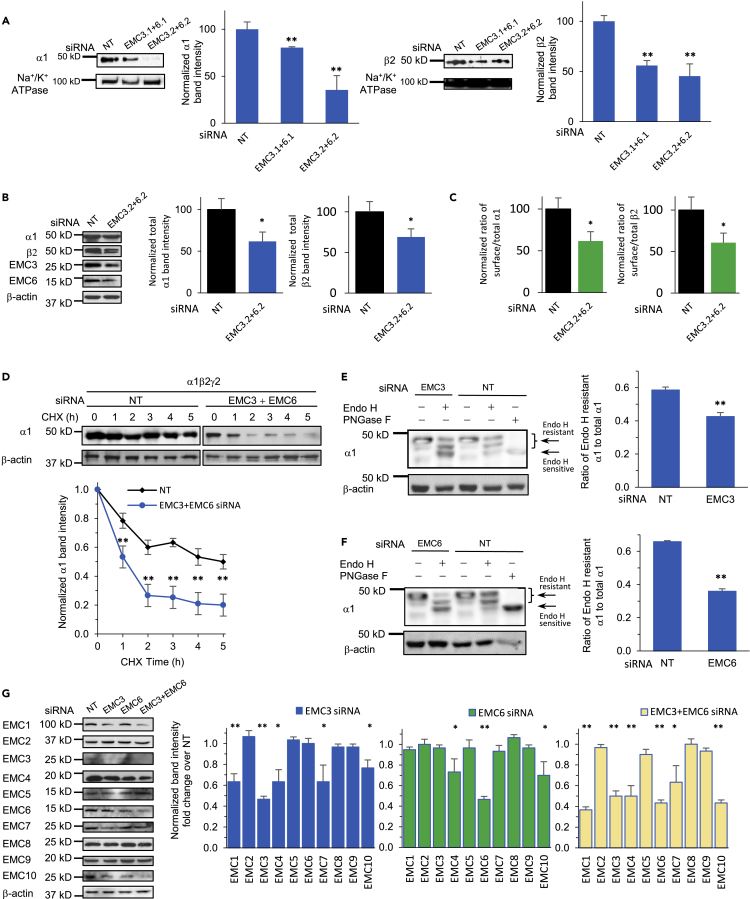
EMC3 and EMC6 promote anterograde trafficking of GABA_A_ receptors (A and B) Significant reduction of cell surface and total α1 and β2 subunits of GABA_A_ receptors was observed when both EMC3 and EMC6 were knocked down. We carried out siRNA transfection in HEK293T cells stably expressing α1β2γ2 GABA_A_ receptors. To test the surface expression of GABA_A_ receptors, biotinylation experiments were performed 48 h after siRNA transfection of both EMC3 and EMC6 (A). Surface proteins were enriched through biotin-neutravidin affinity purification, and western blot analysis was applied to detect surface α1 and β2 subunits. Na^+^/K^+^ ATPase served as loading control of cell surface proteins. To test the total protein expression of GABA_A_ receptors, cells were lysed and total proteins were collected and subjected to SDS-PAGE and western blot analysis (B). β-actin was used as the total protein loading control. Normalized band intensity was shown on the right to the blots (n = 3). (C) The ratio of the surface/total subunits of GABA_A_ receptors was quantified, as a measure of their surface trafficking efficiency. Data was taken from (A) and (B) for the calculation. (D) HEK293T cells stably expressing α1β2γ2 GABA_A_ receptors were transfected with non-targeting siRNA or siRNAs against EMC3 and EMC6. 48 h after transfection, cycloheximide (CHX) (100 μg/mL), a potent protein synthesis inhibitor, was added to cell culture media for the indicated time. Cells were then harvested, and total proteins were subjected to SDS-PAGE and western blot analysis. Quantification of the α1 band intensity was plotted against the incubation time with CHX (n = 3). (E and F) EMC3 and EMC6 promote GABA_A_ receptors’ trafficking from the ER to Golgi as demonstrated through endoglycosidase H (Endo H) digestion. EMC3 or EMC6 siRNA transfection was applied in HEK293T cells stably expressing α1β2γ2 GABA_A_ receptors; 48 h after transfection, proteins were extracted, and subjected to Endo H digestion and western blot analysis. Endo H resistant bands (top two bands in lanes two and 4) represent proteins that have correctly folded in the ER, trafficked to Golgi and fully modified with the *N*-linked complex glycans, thus becoming resistant to Endo H. On the other hand, acting upon proteins remaining in ER, Endo H may remove the high mannose structure after the asparaginyl-*N*-acetyl-D-glucosamine on the α1 subunits, generating Endo H sensitive bands (bottom band in lanes two and 4). The Peptide-N-Glycosidase F (PNGase F) enzyme-treated samples served as a control for unglycosylated α1 subunits (lane 5). Quantification of the ratio of Endo H resistant/total α1 band intensity, as a measure of the trafficking efficiency of α1 subunits, was shown on the right (n = 3). (G) HEK293T cells stably expressing α1β2γ2 GABA_A_ receptors were transfected with non-targeting siRNA or siRNAs against EMC3, EMC6, or both EMC3 and EMC6. 48 h after transfection, cells were harvested, and total proteins were subjected to SDS-PAGE and western blot analysis. Quantification of the normalized individual EMC subunit band intensity was shown on the right panels (n = 3). Each data point is presented as mean ± SEM ∗, p< 0.05; ∗∗, p< 0.01. NT: Non-targeting scrambled siRNA.

In addition, we carried out surface biotinylation experiments to evaluate the effect of the EMC on exogenously expressed human α1β2γ2 GABA_A_ receptors in HEK293T cells. HEK293T cells have been used extensively for the functional expression of neurotransmitter-gated ion channels because HEK293T cells have low or no endogenous expression of such ion channels, and thus precise control of the subtypes can be achieved from the exogenously expressed subunits (Thomas and Smart, 2005). In addition, HEK293T cells are capable of modeling the proteostasis network that orchestrates the folding, assembly, degradation, and trafficking of neurotransmitter-gated ion channels and thus are a suitable vehicle to elucidate the biogenesis pathways of such channels (Wang et al., 2022b). After 48 h of transfection of both EMC3 and EMC6 siRNAs, surface proteins were enriched through biotin-neutravidin affinity purification (see STAR Methods), and western blot analysis was applied to detect α1 and β2 subunits (Figure 3A). Significant reduction of cell surface α1 subunits of GABA_A_ receptors was observed when both EMC3 and EMC6 were knocked down compared to NT, to 78% or 34% when using two sets of siRNAs (Figure 3A, left panel). Similarly, cell surface β2 subunits decreased to 55% or 42% when using two sets of siRNAs (Figure 3A, right panel), when both EMC3 and EMC6 were knocked down. Because knocking down both EMC3 and EMC6 using siRNA set #2 also decreased the total protein level of α1 to 61.8% and that of β2 to 69.2% (Figure 3B), we evaluated whether the reduced surface expression of GABA_A_ receptor subunits was a direct consequence of the lowered total receptor expression level in cells. The ratio of surface/total subunits is commonly used as a measure of their surface trafficking efficiency. Knocking down both EMC3 and EMC6 decreased such a ratio for α1 subunits to 61.5% and β2 subunits to 60.7% (Figure 3C), indicating that EMC3 and EMC6 positively regulate the surface trafficking efficiency of GABA_A_ receptors, which could result from the post-translational effect of EMC3 and EMC6 on the folding and degradation of GABA_A_ receptors.

We hypothesized that knocking down EMC3 and EMC6 would lead to folding and assembly defects of GABA_A_ receptors in the ER and thus their retention in the ER and excessive degradation, causing compromised anterograde trafficking. We evaluated the effect of EMC3 and EMC6 on the degradation kinetics of GABA_A_ receptors using cycloheximide-chase experiments. Remarkably, compared to the non-targeting control, knocking down EMC3 and EMC6 using siRNAs decreased the relative protein abundance of the α1 subunit at each time point during the 5 h chase period after the addition of cycloheximide, a potent protein synthesis inhibitor, in HEK293T cells stably expressing α1β2γ2 GABA_A_receptors (Figure 3D), These results indicated that depleting EMC3 and EMC6 accelerated the protein degradation of GABA_A_ receptors. Therefore, EMC3 and EMC6 positively regulate the protein stability of GABA_A_ receptors in cells.

Furthermore, we determined the effect of EMC3 and EMC6 on GABA_A_ receptors’ trafficking from ER to Golgi through the endoglycosidase H (Endo H) digestion assay. The human α1 subunit of GABA_A_ receptors has two *N*-linked glycosylation sites at N38 and N138. Endo H resistant bands represent glycoproteins, which have correctly folded in the ER, trafficked to the Golgi and fully modified with the *N*-linked complex glycans, thus becoming resistant to Endo H. On the other hand, acting on glycoproteins remaining in ER, Endo H may remove the high mannose structure after the asparaginyl-*N*-acetyl-D-glucosamine on the α1 subunits of GABA_A_ receptors. We carried out EMC3 or EMC6 siRNA transfection in HEK293T cells expressing α1β2γ2 GABA_A_ receptors; 48 h after transfection, proteins were extracted, and subjected to Endo H digestion and western blot analysis (Figures 3E and 3F). The Peptide-N-Glycosidase F (PNGase F) enzyme-treated samples served as a control for unglycosylated α1 subunits (Figures 3E and 3F, lane 5). Two endo H resistant α1 bands were observed, corresponding to singly or doubly glycosylated α1 at N38 and N138 ((Figures 3E and 3F, lane 5, lanes two and 4). Ratio of Endo H resistant α1 to total α1 represents its ER-to-Golgi trafficking efficiency. Such a ratio decreased to 0.43 with EMC3 siRNA (Figure 3E, lane 2), comparing to a ratio of 0.59 of NT (Figure 3E, lane 4). Similarly, the ratio decreased to 0.37 with EMC6 siRNA (Figure 3F, lane 2), comparing to a ratio of 0.65 of NT (Figure 3F, lane 4). Therefore, EMC3 and EMC6 have shown capability of positively regulating the stability of GABA_A_ receptors (Figure 3D) and promoting their anterograde trafficking from the ER to the Golgi post-translationally (Figures 3E and 3F) and onward to the plasma membrane (Figures 3A and 3C).

Because EMC3 and EMC6 are the core subunits of the EMC complex, depleting them was reported to disrupt the formation of the EMC complex in U-2 OS cells (Volkmar et al., 2019). Therefore, we evaluated the effect of EMC3 and EMC6 on the stability of the entire EMC complex, which could contribute to the regulation of GABA_A_ receptor biogenesis in HEK293T cells. Knocking down EMC3 or knocking down both EMC3 and EMC6 using siRNAs reduced the protein levels of EMC1, EMC4, EMC7, and EMC10, which are EMC subunits with transmembrane domains, whereas knocking down EMC6 reduced the protein levels of EMC4 and EMC10 (Figure 3G). The results indicated that depleting EMC3 or EMC6 reduced the protein levels of several other EMC subunits with transmembrane domains, leading to the disruption of the EMC complex. Markedly, EMC3 had a more dramatic effect than EMC6 on the protein levels of other EMC subunits. Therefore, individual EMC subunits could have different levels of capacity to stabilize the EMC, which in turn could contribute to their differentiating influence on the protein levels of GABA_A_ receptors. However, it is still possible that EMC3 and EMC6 could have their distinct functional role on the folding and trafficking of GABA_A_ receptors independent of the formation of the EMC complex.

### Co-immunoprecipitation demonstrated endogenous interactions between EMC3/EMC6 and a number of major neuroreceptors, including GABA_A_ receptors, N-methyl-D-aspartate receptors (NMDARs), and nicotinic acetylcholine receptors (nAChRs)

**Figure 4:**
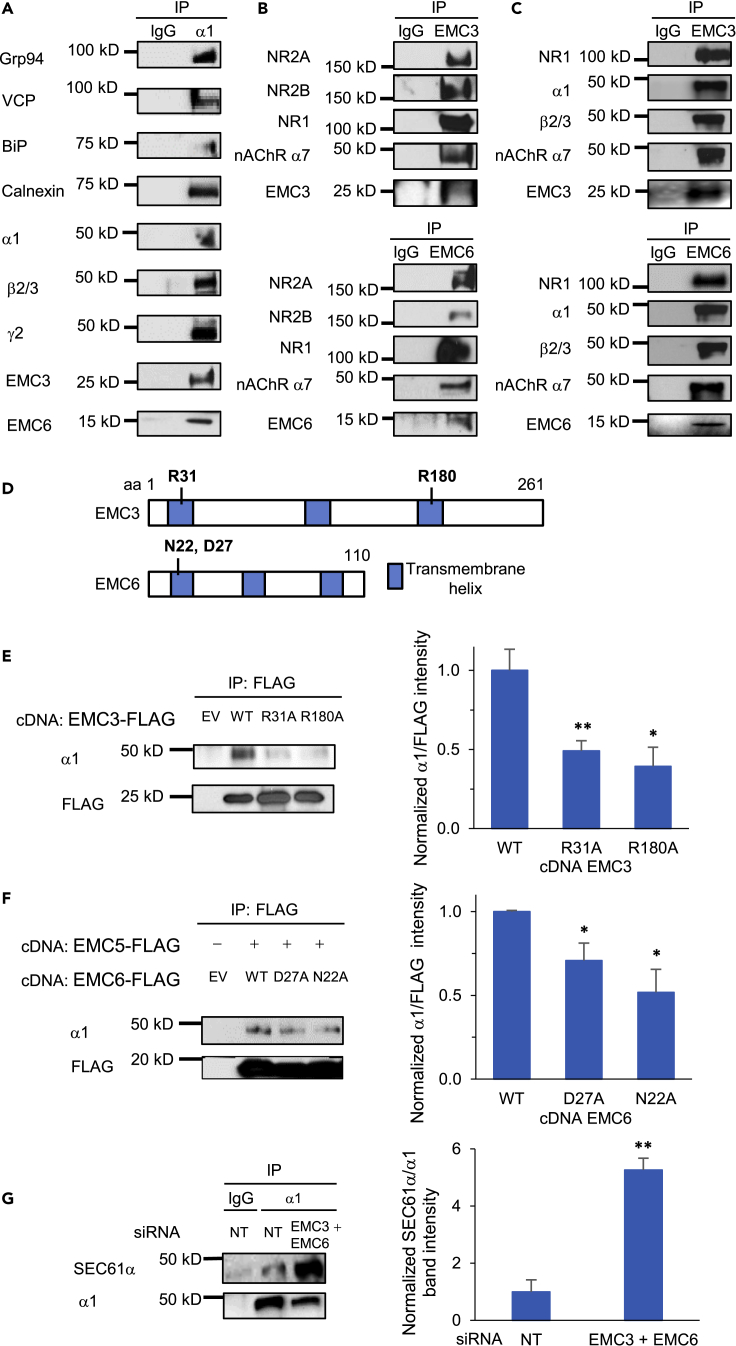
Interactions of EMC3 and EMC6 with neurotransmitter-gated ion channels (A) Co-immunoprecipitation (Co-IP) from primary rat cortical neurons demonstrated endogenous interactions between α1 subunits of GABA_A_ receptors and EMC3, EMC6, and a number of α1-interacting chaperones (BiP and calnexin) and ERAD factors (Grp94 and VCP). Neurons were plated onto 10-cm dishes at a density of one million per dish. At DIV 12, proteins were extracted for Co-IP. IgG was used as a negative control during the immunoprecipitation. n = 3. (B) Co-IP from primary rat cortical neurons demonstrated endogenous interactions between EMC3/EMC6 and a number of ion channels, including N-methyl-D-aspartate receptors (NMDARs, including NR1, NR2A and NR2B subunits) and nicotinic acetylcholine receptors (nAChR α7 subunit). n = 3. (C) Co-IP from mouse cortical homogenates, which were prepared from C57BL/6J mice between 8 and 10 weeks of age, demonstrated endogenous interactions between EMC3/EMC6 and selected ion channels. n = 3. (D) Schematic of the primary sequence of EMC3 and EMC6. R31 and R180 in EMC3 and N22 and D27 in EMC6 were reported to influence the biogenesis of EMC-dependent client proteins. (E) Mutation of R31A or R180A in EMC3 significantly reduced the interaction of EMC3 with GABA_A_ α1 subunits. The cDNAs of FLAG-tagged EMC3, either in the wild type (WT) form or carrying appropriate mutations of R31A or R180A, were transiently transfected in HEK293T cells stably expressing α1β2γ2 GABA_A_ receptors. 48 h after transfection, proteins were extracted from cell lysates and incubated with anti-FLAG M2 magnetic beads. The immuno-purified eluents were separated through SDS-PAGE gel, and western blot analysis was performed to detect α1 subunits and FLAG. Quantification of the band intensity of α1 over FLAG after immunoprecipitation was shown on the right (n = 3). (F) Mutation of D27A or N22A in EMC6 significantly reduced the interaction of EMC6 with GABA_A_R α1 subunits. Transfection of cDNAs was applied similarly as in E, however with co-application of FLAG-tagged EMC5 and EMC6 variants in HEK293T cells stably expressing α1β2γ2 GABA_A_ receptors. Co-IP and visualization of protein bands were carried out the same way as in E as well. Quantification of the band intensity of α1 over FLAG-tagged EMC6 after immunoprecipitation was shown on the right (n = 3). (G) Significant increase of the interaction of SEC61α and α1 subunits of GABA_A_ receptors was observed when both EMC3 and EMC6 were knocked down. We carried out siRNA transfection in HEK293T cells stably expressing α1β2γ2 GABA_A_ receptors; 48 h after transfection, proteins were extracted from cell lysates and incubated with anti-α1 antibody. The immuno-purified eluents were separated through SDS-PAGE gel, and western blot analysis was performed to detect SEC61α and α1 subunits. Quantification of the band intensity of SEC61α over α1 after immunoprecipitation was shown on the right (n = 3). Each data point is presented as mean ± SEM ∗, p< 0.05; ∗∗, p< 0.01. NT: Non-targeting scrambled siRNA; IP: immunoprecipitation; EV: empty vector; WT: wild type.

To facilitate the biogenesis of its client membrane proteins, the EMC is expected to interact with them. Therefore, we continued to understand the interactions between EMC3/6 and GABA_A_ receptors. Rat cortical neurons at DIV 12 were harvested to perform co-immunoprecipitation. Endogenous interactions were confirmed between α1 subunits and EMC3/EMC6 (Figure 4A). In addition, as expected, pulling down α1 subunits led to the detection of β2/3 and γ2 subunits of GABA_A_ receptors, indicating the endogenous interactions within the pentameric receptors. Furthermore, because of the important role of the proteostasis network in orchestrating GABA_A_ receptor biogenesis (Wang et al., 2022b), we observed α1-interacting chaperones, including BiP and calnexin, and ERAD factors, including Grp94 and VCP (Figure 4A). Previously, we demonstrated the role of BiP and calnexin in assisting the folding and the role of Grp94 and VCP in facilitating the degradation of GABA_A_ receptors (Di et al., 2013, 2016; Han et al., 2015a, 2015b).

Furthermore, we evaluated whether the EMC has a more general role in interacting with other major endogenous neurotransmitter-gated ion channels, including nAChRs and NMDA receptors. Both GABA_A_ receptors and nAChRs belong to the Cys-loop superfamily neuroreceptors, sharing a pentameric scaffold (Fu et al., 2016), whereas NMDA receptors are tetramers, consisting of two NR1 subunits and two NR2 subunits (NR2A or NR2B, or both) (Hansen et al., 2021). Dysfunction of these receptors leads to neurological, cognitive and psychiatric brain diseases (Shen et al., 2016a; Steinlein, 2012; Wanamaker et al., 2003). Co-immunoprecipitation experiments demonstrated that pulling down EMC3 or EMC6 resulted in the detection of NR1, NR2A, and NR2B subunits of NMDA receptors as well as α7 subunits of nAChRs in primary cortical neurons (Figure 4B). In addition, mouse cortical homogenates from C57BL/6J mice between 8 and 10 weeks of age, which have developed mature nervous systems, were used to detect the endogenous interactions between EMC3/EMC6 and selected neuroreceptors in physiologically relevant conditions. Consistently, co-immunoprecipitation assay showed that EMC3 and EMC6 interacted with α1 and β2/β3 subunits of GABA_A_ receptors, NR1 subunit of NMDA receptors, and α7 subunit of the nAChRs in cortical homogenates (Figure 4C). These experiments successfully demonstrated the interactions between the EMC and a number of major neuroreceptors in both early and mature stages of development, suggesting that the EMC not only assists folding of GABA_A_ receptors but also potentially plays critical roles for the biogenesis of other multi-pass transmembrane receptors in the CNS.

### Mutating key transmembrane residues of EMC3 and EMC6 significantly impairs their capability of interacting with GABA_A_ receptors

Next, we determined whether the interactions between EMC3/6 and GABA_A_ receptors are through their transmembrane domains. For EMC3, previous literature has shown the importance of positive residues at Arg31 (R31) in transmembrane helix 1 (TM1) and Arg180 (R180) in transmembrane helix 3 (TM3) (Figure 4D), and the absence of such positive residues destabilize post- and co-translational insertion of EMC-dependent substrates, such as squalene synthase and opioid kappa receptor 1 (Pleiner et al., 2020). For that reason, we hypothesized that a neutral residue at such positions would likewise destabilize the interaction between EMC3 and GABA_A_ receptors. FLAG-tagged WT EMC3 or EMC3 carrying appropriate mutations of R31A or R180A were transiently transfected in HEK293T cells stably expressing α1β2γ2 GABA_A_ receptors. The immuno-purified eluents were separated through SDS-PAGE, and western blot analysis was performed to detect α1 subunits and FLAG. As demonstrated by normalized α1 intensity over FLAG, comparing to WT EMC3, significant decrease of the interaction of mutant EMC3 and α1 subunits was observed, to an extent of 0.48 with R31A, or 0.40 with R180A, respectively (Figure 4E). Therefore, the result indicated that a neutral residue replacement at R31 in TM1 and R180 in TM3 impairs EMC3’s capability to interact with GABA_A_ receptors.

Moreover, EMC6 residues in the hydrophilic vestibule are thought to be necessary for the insertion of its substrates (Figure 4D) (Pleiner et al., 2020) Asp27 (D27) in TM1 has been shown with such possibility, and is conserved across several species such as *Homo sapiens, M musculus*, and *Saccharomyces cerevisiae* (Pleiner et al., 2020). In addition, Asn22 (N22) has been identified in TM1 of EMC6 within hydrogen bonding distance of the main chain of EMC5, which contributes to the stabilization of TM1 of EMC6 in the lipid bilayer. Therefore similarly, FLAG-tagged EMC5 and appropriate EMC6 variants were transfected in HEK293T cells stably expressing α1β2γ2 GABA_A_ receptors. Co-expression of EMC5 with EMC6 was necessary because EMC5 is required to stably insert EMC6’s TM1 (Pleiner et al., 2020). Comparing to WT EMC6, its variants led to significant decrease of its interaction with α1 subunits, to an extent of 0.72 with D27A, or 0.55 with N22A, respectively (Figure 4F). The result indicated that D27 and N22 in TM1 play critical roles for EMC6’s interaction with GABA_A_ receptors. Taken together, we showed that the interactions between EMC3/6 and GABA_A_ receptors are dependent on the key charged/polar residues in the TM domains of the EMC, consistent with the role of the EMC as an insertase for multi-pass transmembrane proteins.

Furthermore, because the SEC61 translocon is known to play a crucial role in the insertion of secretory and membrane polypeptides into the ER co-translationally (Skach, 2009) and the EMC coordinates with SEC61 for the insertion of transmembrane proteins (Chitwood et al., 2018; O'Keefe et al., 2021), we investigated the potential orchestration of SEC61 and EMC on the GABA_A_ receptor biogenesis. We carried out siRNA transfection of both EMC3 and EMC6 in HEK293T cells stably expressing α1β2γ2 GABA_A_ receptors. Co-immunoprecipitation experiments showed that significant increase of the interaction of SEC61α and α1 subunits of GABA_A_ receptors was observed when both EMC3 and EMC6 were knocked down, as demonstrated by normalized SEC61α over α1 intensity comparing to NT, to a remarkable extent of 5.3-fold (Figure 4G). Therefore, the result suggested that on depletion of EMC3 and EMC6, GABA_A_ receptors would be routed to the SEC61 translocon for the insertion into the lipid bilayer.

### Overexpression of EMC3 and EMC5/6 restores functional surface expression of disease-associated variants (DAVs) of GABA_A_ receptors

**Figure 5:**
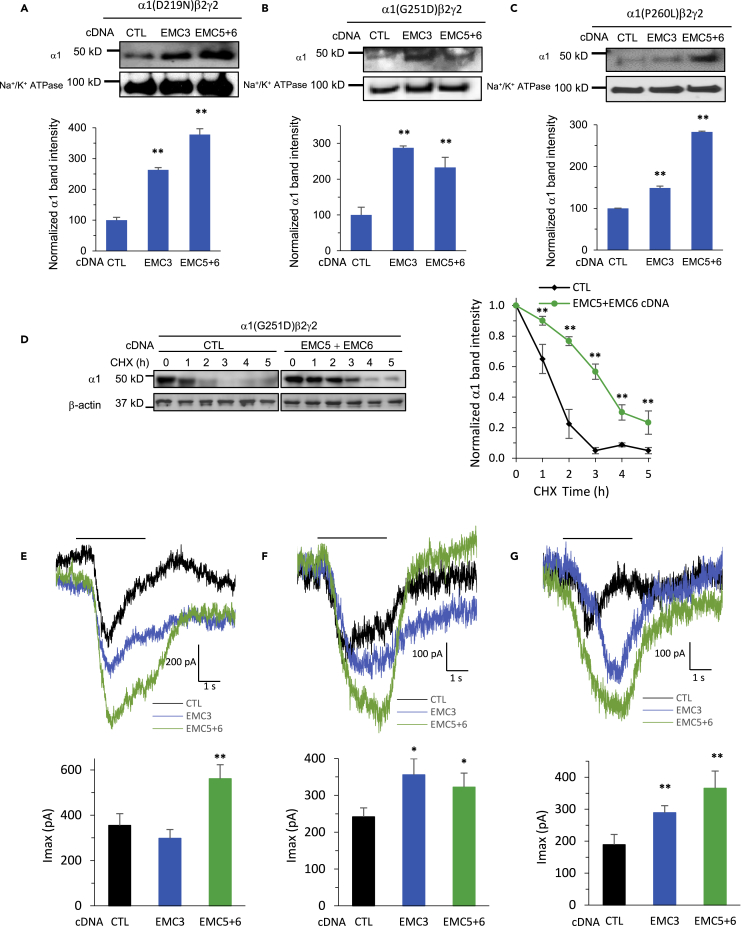
Overexpression of EMC3, and EMC5 and EMC6 restores surface expression and whole-cell currents of disease-associated variants of GABA_A_ receptors (A–C). Overexpression of EMC3 and EMC5/6 increased surface expression of α1 subunits of GABA_A_R in HEK293T cells stably expressing α1(D219N)β2γ2 (A), α1(G251D)β2γ2 (B) and α1(P260L)β2γ2 (C). We carried out cDNA transfection of EMC3, or co-application of EMC5 and EMC6, in corresponding HEK293T cells; 48 h after transfection, surface proteins were enriched through biotin-neutravidin affinity purification, and western blot analysis was applied to detect α1 subunits. Na^+^/K^+^ ATPase served as loading control of cell surface proteins. Normalized surface α1 band intensity was shown below the images (n = 3). (D) HEK293T cells stably expressing α1(G251D)β2γ2 GABA_A_ receptors were transfected with empty vector control (CTL) or EMC5 and EMC6 cDNAs. 48 h after transfection, cycloheximide (CHX) (100 μg/mL), a potent protein synthesis inhibitor, was added to cell culture media for the indicated time. Cells were then harvested, and total proteins were subjected to SDS-PAGE and western blot analysis. Quantification of the α1 band intensity was plotted against the incubation time with CHX (n = 3). (E–G) Increased whole-cell patch-clamping currents of GABA_A_ receptors were recorded in HEK293T cells stably expressing α1(D219N)β2γ2 (E), α1(G251D)β2γ2 (F) and α1(P260L)β2γ2 (G). Transfection of cDNA was applied the same way as in (A–C); 48 h after transfection, patch clamping was performed on the cells with the IonFlux Mercury 16 ensemble plates at a holding potential of −60 mV. GABA (100 μM) was applied for 4 s, as indicated by the horizontal bar above the currents. The peak currents (Imax) were acquired and analyzed by Fluxion Data Analyzer (n = 6 - 10). Each data point is presented as mean ± SEM ∗, p< 0.05; ∗∗, p< 0.01. CTL: Empty vector control sample.

Based on the above-mentioned results, increasing the EMC expression has the promise to enhance forward trafficking and thus surface expression of GABA_A_ receptors, and ultimately their function as neurotransmitter-gated ion channels. To investigate this hypothesis, we evaluated the role of key EMC subunits in epilepsy-associated GABA_A_ receptor variants, which are known to cause subunit misfolding and reduced surface expression, including α1(D219N), α1(G251D), and α1(P260L) (Fu et al., 2022; Han et al., 2015b; Kodera et al., 2016). We carried out cDNA transfection of EMC3 or co-application of EMC5 and EMC6 in HEK293T cells expressing the variants. With EMC3 overexpression, significantly increased surface expression of α1 subunits of GABA_A_ receptors was observed in HEK293T cells stably expressing α1(D219N)β2γ2 (Figure 5A), α1(G251D)β2γ2 (Figure 5B) and α1(P260L)β2γ2 (Figure 5C), to 260%, 285%, and 148% respectively. Similarly, with co-application of EMC5 and EMC6, surface α1 subunits increased to 380%, 235%, and 285% respectively in α1(D219N)β2γ2 (Figure 5A), α1(G251D)β2γ2 (Figure 5B) and α1(P260L)β2γ2 (Figure 5C). Moreover, cycloheximide-chase experiments demonstrated that overexpressing EMC5 and EMC6 substantially slowed down the degradation of α1(G251D) variant in HEK293T cells stably expressing α1(G251D)β2γ2 GABA_A_ receptors (Figure 5D). These results indicated that co-application of EMC5 and EMC6 stabilized the α1(G251D) variant to promote their trafficking to the plasma membrane.

To further evaluate the EMC’s effects on the function of these variants, whole-cell patch-clamping was performed on cells 48 h after transfection using the Fluxion automated patch clamping instrument (see STAR Methods). Increased GABA-induced currents were recorded, as shown in α1(D219N)β2γ2 (Figure 5E), α1(G251D)β2γ2 (Figure 5F) and α1(P260L)β2γ2 (Figure 5G). With EMC3 overexpression, the peak current (Imax) increased from 239 pA to 355 pA and from 185 pA to 285 pA respectively in α1(G251D)β2γ2 (Figure 5F) and α1(P260L)β2γ2 (Figure 5G); no significant change was observed in α1(D219N)β2γ2 (Figure 5E), potentially because of its lesion being in the ER lumen, whereas the EMC acts preferably on the transmembrane domains (Chitwood and Hegde, 2019). Moreover, with co-application of EMC5 and EMC6, Imax increased from 270 pA to 550 pA, 239 pA to 325 pA, and 185 pA to 370 pA respectively in α1(D219N)β2γ2 (Figure 5E), α1(G251D)β2γ2 (Figure 5F) and α1(P260L)β2γ2 (Figure 5G). We observed a modest increase in the peak current compared to the substantial increase in the surface expression of α1 subunit variants because the peak current is also determined by multiple electrophysiological parameters (Sallard et al., 2021). The macroscopic current amplitude at a given time point is the product of the number of open channels and their single channel conductance. The open states of GABA_A_ receptors are also influenced by several kinetic parameters, such as activation, desensitization, and deactivation time scales. In addition, GABA_A_ receptors on the cell surface could have binary forms (with α1 and β2 subunits) in addition to triheteromeric forms (with α1, β2 and γ2 subunits); these two forms have different single channel conductance. Since the cell surface α1 subunits could have different opening probability and single channel conductance, their quantity is not strictly proportional to the observed macroscopic peak current amplitude. Such a discrepancy was also reported in GABA_A_ receptors containing ρ1 variants (Hannan and Smart, 2018). Nonetheless, these results indicated that overexpression of EMC3 or EMC5/6 is sufficient to enhance the function of DAVs of GABA_A_ receptors.

## Discussion

In this study, we systemically examined the effects of all 10 individual subunits of the EMC complex on regulating endogenous GABA_A_ receptor protein expression. Significant reduction of the protein levels of GABA_A_ receptor subunits, including α1, β2/β3, and γ2 subunits, and GABA-induced currents were observed from knocking down EMC3 and EMC6 in neurons (Figures 1 and 2). Knocking down EMC3 or EMC6 reduced the protein levels of several other EMC subunits with transmembrane domains in HEK293T cells (Figure 3G), consistent with the report that depleting core EMC subunits disrupts the EMC complex in U-2 OS cells (Volkmar et al., 2019). It appears that depleting EMC3 downregulated more EMC subunits than depleting EMC6. Therefore, on one hand, individual EMC subunits could stabilize the EMC complex to a different extent, causing their differentiating influence on the protein levels of GABA_A_ receptors. On the other hand, because overexpressing EMC3 or EMC5/6 was sufficient to increase the functional surface expression of several GABA_A_ receptor variants (Figure 5), it is also possible that EMC3 and EMC6 could execute their functional role on GABA_A_ receptor folding and trafficking independent of the formation of the EMC complex, which would suggest a subunit-specific contribution of the EMC in regulating the biogenesis of a multi-pass transmembrane protein. Intriguingly, consistent results have been reported, showing that the EMC3-EMC6 fusion protein is sufficient to insert the mitochondrial protein Cox2 and nuclear encoded inner membrane proteins (Güngör et al., 2022), suggesting that certain EMC subunits are capable of carrying out the membrane insertase function. EMC3 is homologous to known membrane protein insertases Oxa1 family proteins (Volkmar and Christianson, 2020; Wideman, 2015), and depletion of EMC3 results in ER stress and activates the unfolded protein response (Huang et al., 2021; Tang et al., 2017). Moreover, EMC3 was reported to coordinate the assembly of lipids and proteins required for surfactant synthesis (Tang et al., 2017), maintain differentiation and function of intestinal exocrine secretory lineages (Huang et al., 2021), and play a critical role in mammalian retinal development (Cao et al., 2021; Zhu et al., 2020). In parallel, in addition to its role in regulating the biogenesis of acetylcholine receptors in *C. elegans* (Richard et al., 2013), EMC6 was reported to regulate the autophagosome formation, and knocking down EMC6 impaired autophagy (Li et al., 2013). Moreover, the important role of EMC6 as a therapeutic target for cancer and pancreatic inflammatory diseases has been increasingly recognized (Shen et al., 2016b; Tan et al., 2020; Wang et al., 2017; Xiao et al., 2021). Nonetheless, the potential subunit-specific contribution of the EMC to the membrane protein quality control merits further investigation.

**Figure 6:**
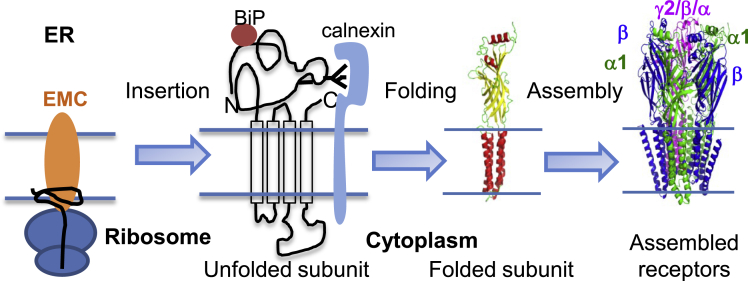
Working model of the role of the EMC on the biogenesis of GABA_A_ receptors in the ER The EMC facilitates the insertion of the transmembrane domains of GABA_A_ receptor subunits into the lipid bilayer. ER chaperones, such as BiP and calnexin, promote the folding of the ER luminal domains of GABA_A_ receptors for subsequent assembly into pentameric receptors in the ER.

Our data support a working model about the role of the EMC in GABA_A_ biogenesis in the ER (Figure 6). The EMC, including EMC3 and EMC6, interacts with the nascent subunits of GABA_A_ receptors through the transmembrane domains. Mechanistic studies revealed that R31 in TM1 and R180 in TM3 of EMC3, and D27 and N22 in TM1 of EMC6 are essential residues for EMC’s interaction with GABA_A_ receptors (Figures 4D–4F), consistent with the structural work of the EMC (Pleiner et al., 2020). Therefore, the EMC facilitates the insertion of the transmembrane domains of GABA_A_ receptor subunits into the lipid bilayer. Subsequently, molecular chaperones, such as BiP and calnexin, promote the productive folding of GABA_A_ receptors (Di et al., 2013; Han et al., 2015b). After the proper assembly of the pentameric receptors on the ER membrane, GABA_A_ receptors engage the trafficking factors, such as LMAN1 (Fu et al., 2019), en route to the Golgi and onward to the plasma membrane. Consistently, we demonstrated that EMC3 and EMC6 promote the anterograde trafficking of GABA_A_ receptors (Figure 3). Here, we focused on GABA_A_ receptor subtypes in the synaptic sites containing the α1 subunit. Given the substantial diversity of GABA_A_ receptor subunits (α1-6, β1-3, γ1-3, δ, ε, θ, π, and ρ1-3), numerous subtypes of GABA_A_ receptors containing the combination of subunits can form either in the synaptic sites to trigger fast, transient phasic inhibition or in the extrasynaptic sites to generate persistent tonic inhibition (Farrant and Nusser, 2005). The α1-α3 subunits are primarily synaptic, whereas α4, α6, and δ subunits are predominantly extrasynaptic (Lorenz-Guertin and Jacob, 2018). Tonic receptors have high affinity to GABA to respond to its “spill over” with low conductance. Since synaptic receptors and extrasynaptic receptors have different subunit composition and functional characteristics, it would be of great interest to determine whether the EMC complex plays similar roles in regulating the biogenesis of phasic and tonic GABA_A_ receptors in future studies. Furthermore, since EMC3 and EMC6 interact with major endogenous neurotransmitter-gated ion channels in primary neurons, including Cys-loop receptors and glutamate receptors (Figures 4A–4C), the EMC could have a general role in the CNS. It would be of great interest to identify the endogenous interactomes of the EMC subunits, such as EMC3 and EMC6, as a way to determine their client membrane proteins in the CNS in the future.

Loss of function of inhibitory GABA_A_ receptors is one of the primary causes of genetic epilepsy. Recent advance in genetics has identified over 150 epilepsy-associated variants in the major subunits (α1, β2, β3, and γ2) of GABA_A_ receptors (Absalom et al., 2022; Fu et al., 2022; Hernandez and Macdonald, 2019; Hirose, 2014). Despite the development of numerous anti-seizure drugs, about one-third epilepsy patients are resistant to current drug treatment (Rincon et al., 2021), and many of them have genetic causes (Smolarz et al., 2021). Therefore, there is an urgent need to develop new therapeutic strategy to treat epilepsy, especially drug-resistant epilepsy. Because one major disease-causing mechanism for loss of function of GABA_A_ receptors is their reduced trafficking to the plasma membrane, one promising approach is to adapt the proteostasis network to restore their surface trafficking and thus function (Di et al., 2013, 2021; Fu et al., 2018; Han et al., 2015a, 2015b). Recently, we showed that pharmacological activation of the ATF6 arm of the unfolded protein response using small molecules AA147 and AA263 restored the functional surface expression of misfolding-prone GABA_A_ variants containing α1(D219N), γ2(R82Q), or γ2(R177G) by increasing the protein levels of pro-folding chaperones, such as BiP, and the interactions between these chaperones and GABA_A_ variants; importantly, ATF6 activators are more selective for folding defective variants compared to wild type receptors (Wang et al., 2022a). Furthermore, such a selectivity was also reported when other small molecule proteostasis regulators, such as verapamil, dinoprost, and dihydroergocristine, were applied to rescue misfolding-prone GABA_A_ variants (Di et al., 2021; Han et al., 2015b), indicating that adapting the proteostasis network has the promise to achieve selectivity for disease-associated variants. Here, we demonstrated that overexpression of EMC3 and EMC5/6 enhances the functional surface expression of a number of folding-deficient GABA_A_ receptor variants (Figure 5). Because EMC3 and EMC5/6 interact with both inhibitory GABA_A_ receptors and excitatory NMDARs and nAChRs (Figures 4A–4C), the EMC complex has the potential to regulate the biogenesis of a number of neuroreceptors that regulate excitation-inhibition balance. Therefore, in order to evaluate the suitability of the EMC as the therapeutic target to rescue pathogenic GABA_A_ receptors, future research is needed to determine whether activating the EMC is capable of achieving selectivity to correct the function of misfolding-prone GABA_A_ receptor variants over wild type receptors.

### Limitations of the study

The current study did not elucidate how the EMC coordinates with other proteostasis network components to orchestrate the biogenesis of GABA_A_ receptors in the ER. In addition, the potential general role of the EMC in regulating the protein quality control of neurotransmitter-gated ion channels in the CNS needs to be further investigated.

## STAR★Methods

### Key resources table

**Table:** 

REAGENT or RESOURCE	SOURCE	IDENTIFIER
**Antibodies**		

Mouse monoclonal anti-GABA_A_R α1 (clone BD24)	Millipore	MAB339; RRID:AB_2108828
Mouse monoclonal anti-GABA_A_R β2/3 (clone 62-3G1)	Millipore	05-474; RRID:AB_309747
Rabbit monoclonal anti-GABA_A_R α1	Synaptic systems	224203; RRID:AB_2232180
Rabbit polyclonal anti-GABA_A_R γ2	Synaptic systems	224003; RRID:AB_2263066
Rabbit polyclonal anti-GABA_A_R α1	R&D systems	PPS022; RRID:AB_2294487
Fluorescent anti-β-actin antibody Rhodamine	Biorad	12004163; RRID:AB_2861334
Mouse monoclonal anti-β-actin	Sigma Aldrich	A1978; RRID:AB_476692
Mouse monoclonal anti-FLAG (clone M2)	Sigma Aldrich	F1804; RRID:AB_262044
Rabbit polyclonal anti-calnexin	Enzo life sciences	ADI-SPA-860-F; RRID:AB_11178981
Rat monoclonal anti-Grp94 (clone 9G10)	Enzo life sciences	ADI-SPA-850-F; RRID:AB_11179746
Rabbit polyclonal anti-VCP	Abgent	AP6920b; RRID:AB_1968401
Rabbit polyclonal anti-SEC61α	Proteintech	24935-1-AP; RRID:AB_2879807
Rabbit polyclonal anti-Grp78	Abcam	ab21685; RRID:AB_2119834
Rabbit monoclonal anti-Na^+^/K^+^-ATPase	Abcam	ab76020; RRID:AB_1310695
Rabbit polyclonal anti-EMC1	Abcepta	AP10226b; RRID:AB_10817224
Rabbit polyclonal anti-EMC2	Proteintech	25443-1-AP; RRID:AB_2750836
Rabbit polyclonal anti-EMC3	Abcepta	AP5782a; RRID:AB_10816577
Rabbit polyclonal anti-EMC4	Abcepta	AP14717a; RRID:AB_11137185
Rabbit polyclonal anti-EMC5	Pierce	PA5-56905; RRID:AB_2644014
Rabbit polyclonal anti-EMC6	Pierce	PA5-107119; RRID:AB_2817835
Rabbit polyclonal anti-EMC7	Pierce	PA5-52688; RRID:AB_2641011
Mouse monoclonal anti-EMC8	Proteintech	66547-1-IG; RRID:AB_2881909
Rabbit polyclonal anti-EMC9	Abcepta	AP5632b; RRID:AB_10821215
Rabbit polyclonal anti-EMC10	Abcepta	AP5188a; RRID:AB_10663060
Rabbit monoclonal anti-NR1 antibody	Abcam	ab109182; RRID:AB_10862307
Rabbit monoclonal anti-NR2A	Abcam	ab124913; RRID:AB_10975154
Rabbit monoclonal anti-NR2B	Abcam	Ab183942; RRID:AB_2889878
Rabbit polyclonal anti-nAChR α7	Abcam	ab182442
Goat anti-Mouse IgG (H + L) Secondary Antibody, HRP	Invitrogen	31430; RRID:AB_228307
Goat anti-Rabbit IgG (H + L) Secondary Antibody, HRP	Invitrogen	31460; RRID:AB_228341
Goat anti-Rat IgG (H + L) Secondary Antibody, HRP	Invitrogen	31470; RRID:AB_228356
Alexa Fluor 594-conjugated goat-anti-rabbit secondary antibody	Invitrogen	A11037; RRID:AB_2534095
Alexa Fluor 594-conjugated goat-anti-mouse secondary antibody	Invitrogen	A11032; RRID:AB_2534091

**Bacterial and virus strains**		

MAX Efficiency DH5α competent cells	Invitrogen	18258012
psPAX2	Addgene	12260
pMD2.G	Addgene	12259
scrambled siRNA lentivector	Abmgood	LV015-G
EMC3-set of four siRNA lentivectors (rat)	Abmgood	468690960395
EMC6-set of four siRNA lentivectors (rat)	Abmgood	471140960395

**Biological samples**		

Sprague Dawley rat E18 brain cortex tissue	BrainBits	SDECX

**Chemicals, peptides, and recombinant proteins**		

Dulbecco’s Modified Eagle Medium	Fisher Scientific	10-013-CV
Dulbecco’s Phosphate Buffered Saline	Fisher Scientific	SH3002803
Fetal Bovine Serum (FBS), heat-inactivated	Fisher Scientific	SH3039603HI
HEK293 SFM II	Invitrogen	11686-029
Penicillin-Streptomycin	Fisher Scientific	SV30010
Trypsin protease	Fisher Scientific	SH3023601
Accutase	Sigma Aldrich	A6964
HEPES	Invitrogen	15630-080
Neurobasal Medium	Invitrogen	21103049
B-27 supplement (50X)	Invitrogen	17504044
GlutaMAX Supplement	Invitrogen	35050061
DAPI (4′,6-Diamidino-2-Phenylindole, Dihydrochloride)	Invitrogen	D1306
Opti-MEM Reduced Serum Medium	Invitrogen	31985070
TansIT-2020 Transfection Reagent	Mirus Bio	MIR 5400
HiPerfect Transfection Reagent	Qiagen	301707
poly-D-lysine	Sigma Aldrich	P6407
Poly-L-lysine	Fisher Scientific	ICN15017710
Laminin	Sigma Aldrich	L2020
Ara-C hydrochloride	Sigma Aldrich	C6645
Lenti-X concentrator	Takara Bio	631231
G418 sulfate	Enzo Life Sciences	ALX-380-013-G005
DMSO	Fisher Scientific	BP231100
γ-Aminobutyric acid	Sigma Aldrich	A2129
Cycloheximide	Enzo Life Sciences	ALX-380-269-G001
Protein A/G plus-agarose beads	Santa Cruz	SC-2003
Normal mouse IgG	Santa Cruz	SC-2025
anti-FLAG M2 magnetic beads	Sigma Aldrich	M8823
Sulfo-NHS-SS-Biotin	APExBio	A8005
N-ethylmaleimide (NEM)	ThermoFisher Pierce	PI23030
NeutrAvidin agarose resin	ThermoFisher Pierce	PI29200
Complete mini EDTA-free protease inhibitor cocktail	Roche	4693159001
n-Dodecyl-B-D-maltoside (DDM)	GoldBio	DDM5
Endo H_f_ enzyme	NEB	P0703L
Peptide-N-Glycosidase F (PNGase F) enzyme	NEB	P0704L
40% acrylamide/Bis Solution 29:1	Biorad	1610146
2x Laemmli sample buffer	Biorad	1610737
4x Laemmli sample buffer	Biorad	1610747
Super-Signal West Pico PLUS Chemiluminescent Substrate	ThermoFisher Pierce	34578
Super-Signal West Femto Maximum Sensitivity Substrate	ThermoFisher Pierce	34096

**Critical commercial assays**		

MicroBCA protein assay	ThermoFisher Pierce	23235
QuikChange II site-directed mutagenesis Kit	Agilent Genomics	200523
qPCR lentivirus titration kit	Abmgood	LV900

**Experimental models: Cell lines**		

HEK293T (donor sex: female)	ATCC	CRL-3216; RRID:CVCL_0063
GT1-7 Mouse Hypothalamic Neuronal Cell Line	Millipore	SCC116; RRID:CVCL_0281

**Experimental models: Organisms/strains**		

C57BL/6J mice	The Jackson Laboratory	RRID:IMSR_JAX:000664

**siRNA**		

siRNA non-targeting control	Dharmacon	D-001810-01-20
EMC1.1	Dharmacon	J-059370-09-0005
EMC1.2	Dharmacon	J-059370-10-0005
EMC2.1	Dharmacon	J-049743-09-0005
EMC2.2	Dharmacon	J-049743-10-0005
EMC3.1	Dharmacon	J-056059-09-0005
EMC3.2	Dharmacon	J-056059-11-0005
EMC4.1	Dharmacon	J-046351-09-0005
EMC4.2	Dharmacon	J-046351-10-0005
EMC5.1	Dharmacon	J-041149-09-0005
EMC5.2	Dharmacon	J-041149-11-0005
EMC6.1	Dharmacon	J-047425-10-0005
EMC6.2	Dharmacon	J-047425-12-0005
EMC7.1	Dharmacon	J-051219-09-0005
EMC7.2	Dharmacon	J-051219-11-0005
EMC8.1	Dharmacon	J-046488-09-0005
EMC8.2	Dharmacon	J-046488-10-0005
EMC9.1	Dharmacon	J-046998-09-0005
EMC9.2	Dharmacon	J-046998-10-0005
EMC10.1	Dharmacon	J-041588-11-0005
EMC10.2	Dharmacon	J-041588-12-0005

**Recombinant DNA**		

Plasmid: human GABA_A_R-α1 (pCMV6)	OriGene Technologies	RC205390
Plasmid: human GABA_A_R-β2 (pCMV6)	OriGene Technologies	RC216424
Plasmid: human GABA_A_R-γ2 (pCMV6)	OriGene Technologies	RC209260
Plasmid: pCMV6 Entry vector	OriGene Technologies	PS100001
Plasmid: EMC3	GenScript	OHu03021D
Plasmid: EMC3-R31A	This paper	N/A
Plasmid: EMC3-R180A	This paper	N/A
Plasmid: EMC5	OriGene Technologies	RC207046
Plasmid: EMC6	OriGene Technologies	RC215548
Plasmid: EMC6-D27A	This paper	N/A
Plasmid: EMC6-N22A	This paper	N/A

**Software and algorithms**		

ImageJ	National Institutes of Health	https://imagej.nih.gov/ij/
Origin	Origin Lab	https://www.originlab.com/
Automatic patch clamping	Ionflux Mercury16	https://www.fluxionbio.com/ionflux-mercury-automated-patch-clamp

### Resource availability

#### Lead contact

Further information and requests for resources and reagents should be directed to and will be fulfilled by the Lead Contact, Ting-Wei Mu (tingwei.mu@case.edu).

#### Materials availability

All plasmids generated in this study will be made available on request but we may require a payment and/or a completed Materials Transfer Agreement.

### Experimental model and subject details

#### Cell lines

HEK293T cells (#CRL-3216, donor sex: female) were obtained from ATCC. GT1-7 cells (catalog #: SCC116) were obtained from Millipore. Cells were maintained in Dulbecco’s Modified Eagle Medium (DMEM) with 10% heat-inactivated fetal bovine serum (FBS) and 1% Penicillin-Streptomycin at 37°C in 5% CO_2_.

### Method details

#### Reagents

The pCMV6 plasmids containing human GABA_A_ receptor α1 (Uniprot no. P14867-1), β2 (isoform 2, Uniprot no. P47870-1), γ2 (isoform 2, Uniprot no. P18507-2) subunits, and pCMV6 Entry Vector plasmid (pCMV6-EV) were obtained from OriGene. The human FLAG-tagged EMC3 plasmid was purchased from GenScript (catalog #: OHu03021D). The human FLAG-tagged EMC5 plasmid (catalog #: RC207046) and FLAG-tagged EMC6 plasmid (catalog #: RC215548) were obtained from OriGene. The mutations GABRA1-D219N, GABRA1-G251D, GABRA1-P260L, EMC3-R31A, EMC3-R180A, EMC6-D27A, and EMC6-N22A were constructed using QuikChange II site-directed mutagenesis Kit (Agilent Genomics, catalog #: 200523). All cDNA sequences were confirmed by DNA sequencing.

The following small interfering RNA (siRNA) duplexes were obtained from Dharmacon: EMC1.1 (J-059370-09-0005), EMC1.2 (J-059370-10-0005), EMC2.1 (J-049743-09-0005), EMC2.2 (J-049743-10-0005), EMC3.1 (J-056059-09-0005), EMC3.2 (J-056059-11-0005), EMC4.1 (J-046351-09-0005), EMC4.2 (J-046351-10-0005), EMC5.1 (J-041149-09-0005) EMC5.2 (J-041149-11-0005), EMC6.1 (J-047425-10-0005), EMC6.2 (J-047425-12-0005), EMC7.1 (J-051219-09-0005), EMC7.2 (J-051219-11-0005), EMC8.1 (J-046488-09-0005), EMC8.2 (J-046488-10-0005), EMC9.1 (J-046998-09-0005), EMC9.2 (J-046998-10-0005), EMC10.1 (J-041588-11-0005), EMC10.2 (J-041588-12-0005), human EMC3.1 (J-010715-17-0005), human EMC3.2 (J-010715-18-0005), human EMC6.1 (J-014711-19-0005), human EMC6.2 (J-014711-20-0005), and Non-Targeting (NT) siRNA (D-001810-01-20), which was used as a negative control. The designation of EMCn.1 and EMCn.2 (n = 1 to 10) indicates two distinct siRNA sequences against each EMC subunit.

#### Antibodies

The rabbit polyclonal EMC1 antibody (catalog #: AP10226b), rabbit polyclonal EMC3 antibody (catalog #: AP5782a), rabbit polyclonal EMC4 antibody (catalog #: AP14717a), rabbit polyclonal EMC9 antibody (catalog #: AP5632b), and rabbit polyclonal EMC10 antibody (catalog #: AP5188a) were from Abcepta. The rabbit polyclonal EMC2 antibody (catalog #: 25443-1-AP) and mouse monoclonal EMC8 antibody (catalog #: 66547-1-IG) were obtained from Proteintech. The rabbit polyclonal EMC5 antibody (catalog #: PA5-56905), rabbit polyclonal EMC6 antibody (catalog #: PA5-107119), and rabbit polyclonal EMC7 antibody (catalog #: PA5-52688) were from Pierce.

The mouse monoclonal anti-GABA_A_ α1 subunit antibody (clone BD24) (catalog #: MAB339) and mouse monoclonal anti-GABA_A_ β2/β3 antibody (catalog #: 05-474) were obtained from Millipore. The rabbit monoclonal anti-GABA_A_ α1 subunit antibody (catalog #: 224203) and rabbit polyclonal anti-GABA_A_ γ2 antibody (catalog #: 224003) were obtained from Synaptic systems. The rabbit polyclonal anti-GABA_A_ α1 antibody came from R&D systems (catalog #: PPS022). The mouse monoclonal anti-β-actin antibody (catalog #: A1978) and mouse monoclonal FLAG antibody (catalog #: F1804) came from Sigma Aldrich. The fluorescent anti-β-actin antibody Rhodamine came from Biorad (catalog #: 12004163). The rabbit polyclonal anti-calnexin (catalog #: ADI-SPA-860-F) and rat polyclonal anti-Grp94 (catalog #: ADI-SPA-850-F) antibodies were purchased from Enzo Life Sciences. The rabbit polyclonal anti-VCP (catalog #: AP6920b) antibody was obtained from Abgent. The rabbit polyclonal anti-SEC61α antibody was obtained from Proteintech (catalog #: 24935-1-AP). The rabbit polyclonal anti-Grp78 antibody (catalog #: ab21685), rabbit monoclonal anti-NR1 antibody (catalog #: ab109182), rabbit monoclonal anti-NR2A antibody (catalog #: ab124913), rabbit monoclonal anti-NR2B antibody (catalog #: ab183942), and rabbit polyclonal anti-nAChR α7 antibody (catalog #: ab182442), and sodium potassium ATPase antibody (catalog #: ab76020) were purchased from Abcam.

The secondary antibodies used from Invitrogen included: HRP-conjugated goat-anti-rabbit (catalog #: 31460), goat-anti-mouse (catalog #: 31430), and goat-anti-rat (catalog #: 31470), and Alexa Fluor 594-conjugated goat-anti-rabbit (catalog #: A11037) and goat-anti-mouse (catalog #: A11032).

#### Cell culture and transfection

HEK293T cells (catalog #: CRL-3216) were obtained from ATCC, and GT1-7 cells (catalog #: SCC116) were obtained from Millipore. Cells were maintained in Dulbecco’s Modified Eagle Medium (DMEM) (Fisher, catalog #: SH3024301) with 10% heat-inactivated fetal bovine serum (ThermoFisher, catalog #: SH3039603HI) and 1% Penicillin-Streptomycin (Hyclone, catalog #: sv30010) at 37°C in 5% CO_2_. Cells were grown in 6-well plates or 10-cm dishes and allowed to reach ∼70% confluency before transient transfection using TransIT-2020 (Mirus, catalog #: MIR 5400), or siRNA treatment (50 nM) using the HiPerfect Transfection Reagent (Qiagen, catalog #: 301707) according to the manufacturer’s instruction. A second siRNA transfection was performed 24 h after the first siRNA treatment to increase knockdown efficiency. Forty-eight hours after transfection, cells were harvested for further analysis.

Stable cell lines for α1β2γ2, α1(D219N)β2γ2, α1(G251D)β2γ2, and α1(P260L)β2γ2 were generated using the G-418 selection method. Briefly, cells were transfected with α1:β2:γ2 (1:1:1), α1(D219N):β2:γ2 (1:1:1), α1(G251D):β2:γ2 (1:1:1) or α1(P260L):β2:γ2 (1:1:1) plasmids, selected in DMEM supplemented with 0.8 mg/mL G418 (Enzo Life Sciences) for 10 days, and then maintained in DMEM supplemented with 0.4 mg/mL G418. G-418 resistant cells were used for experiments.

#### Western blot analysis

To harvest total proteins, cells were washed with Dulbecco’s phosphate-buffered saline (DPBS) (Fisher, catalog #: SH3002803). Trypsin (0.05%) (Fisher, catalog #: SH30236.01) was added to lift the cells, and DMEM was added to harvest cells from the dish and pipette into a centrifuge tube. Cells were spun down for 3 min at 1000 rpm and then DMEM was removed while avoiding the pellet. Cell pellets were washed with DPBS and centrifuged again at 1000 rpm for 3 min. The DPBS was removed, and pellets were stored on ice during transport to a −80°C freezer. Cells were lysed with lysis buffer (50 mM Tris, pH 7.5, 150 mM NaCl, and 2 mM n-Dodecyl-B-D-maltoside (DDM) (GoldBio, catalog #: DDM5)) supplemented with complete protease inhibitor cocktail (Roche). Cells were vortexed for 30 s followed by ultrasonication for 30 s for three times. Then they were centrifuged at 15,000 × *g*, 4°C for 10 min to obtain the supernatant as total proteins. The protein concentration was measured according to Thermo Fisher MicroBCA kit protocol. Cell lysates were loaded with Laemmli sample buffer (Biorad, catalog #: 1610747) with β-mercaptoethanol (1:10 v/v) and separated through SDS-PAGE gel.

Before proceeding to western blot, gels were made ranging from 8% to 20% resolving gel depending on the size of the protein with 4% stacking gel on top. To run SDS-PAGE gel electrophoresis, gels were place in a holding voltage cassette and submerged in the running buffer, which contained the following: 25 mM Tris (Sigma, catalog #: T1503), 192 mM Glycine (Sigma, catalog: # 8898), and 0.1% (w/v) of sodium dodecyl sulfate (SDS, Biorad, catalog #: 1610302). Protein ladder was added (Biorad, catalog #: 1610395). Gels were run at 10 min at 100 volts until the samples passed the stacking gel and were uniformly aligned. For the remaining time of 45 min to 1 h, gels were run at 150 V. After running samples to sufficient molecular weight, gels were transferred at 100 V for 1 h to a nitrocellulose membrane. The transfer buffer contained the following: 25 mM Tris (Sigma, catalog #: T1503), 192 mM Glycine (Sigma, catalog: # 8898), 20% (v/v) of methanol (Fisher Chemical, catalog #: A452-4). After the transfer, membranes were washed briefly in TBS-T, which contained the following: 20 mM Tris (Sigma, catalog #: T1503), 150 mM NaCl (Sigma, catalog #: S7653), pH 7.6, and 0.1% (v/v) Tween20 (Sigma, catalog #: P7949-500mL). They were further incubated in 5% non-fat milk powder (Nestle Carnation, catalog #: 43875) in TBS-T for 30 min to 2 h. Following the blocking step, the membranes were incubated in 1% milk with the primary antibody added starting at 1:1000 dilution and adjusted accordingly on subsequent runs. The following day, the membrane was washed 3 times with TBS-T for 10 min each and incubated with their secondary antibody (1:10,000) for 1 h. This was followed by 3 more washed with TBS-T. Afterward gels were exposed with Pico PLUS (catalog #: 34578) or Femto (catalog #: 34096) SuperSignal West chemiluminescent substrates from Thermo Scientific for three minutes. After using different exposure times to get optimal images, results were analyzed to quantify band intensity using ImageJ software from the NIH.

#### Co-immunoprecipitation (Co-IP)

Cell lysates (500 μg) were pre-cleared with 30 μl of protein A/G plus-agarose beads (Santa Cruz, catalog #: sc-2003) and 1 μg of normal mouse IgG antibody (Santa Cruz, catalog #: sc-2025) for 1 h at 4°C to remove nonspecific binding proteins. The pre-cleared cell lysates were incubated with 2.0 μg of mouse anti-α1 antibody for 1 h at 4°C, and then with 30 μl of protein A/G plus agarose beads overnight at 4°C. For FLAG-tagged proteins, the pre-cleared cell lysates were incubated with 30 μl of anti-FLAG M2 magnetic beads (Sigma, catalog #: M8823-5 mL) overnight at 4°C. IgG serves as negative control. The beads were collected by centrifugation at 8000 × g for 30 s or using a magnet separator (Promega), and washed three times with lysis buffer. The complex was eluted by incubation with 30 μl of Laemmli sample loading buffer in the presence of β-mercaptoethanol. The immuno-purified eluents were separated through SDS-PAGE gel, and western blot analysis was performed.

#### Lentivirus transduction in rat cortical neurons

Lentivirus were generated from transiently transfected HEK293T cells and collected after 60 h from the media. Briefly, HEK293T cells were grown in 10-cm dishes and allowed to reach ∼70% confluency before transient transfection using TransIT-2020 (Mirus), according to the manufacturer’s instruction. The following plasmid (6 μg) was added to 10-cm dishes: EMC3-set of four siRNA lentivectors (rat, Abmgood, catalog #: 468690960395), or EMC6-set of four siRNA lentivectors (rat, Abmgood, catalog #: 471140960395), or scrambled siRNA lentivector (Abmgood, catalog #: LV015-G) as the control. Additionally, to form the lentivirus, the following packaging and envelop plasmids were added to all of the 10-cm dishes as well: psPAX2 (6 μg) and pMD2.G (0.75 μg). psPAX2 (Addgene plasmid # 12260; http://n2t.net/addgene:12260; RRID:Addgene_12260) and pMD2.G (Addgene plasmid # 12259; http://n2t.net/addgene:12259; RRID:Addgene_12259) were a gift from Didier Trono. Media was changed after 8 h; after 52 additional h, media was harvested and passed through 0.45 μm filter (Advantec, catalog #: 25CS045AS) to collect the lentivirus. Furthermore, the lentivirus were concentrated using Lenti-X concentrator (Takara Bio, Catalog #: 631231) and quantified with the qPCR lentivirus titration kit (Abmgood, catalog #: LV900) according to the manufacturer’s instruction, and saved to −80°C for neuron cells transduction.

Sprague Dawley rat E18 brain cortex tissues were obtained from BrainBits, with provided Hibernate EB (HEB) and NbActiv1 media (Springfield, IL). Prior to plating cells, 10-cm dishes or coverslips (Fisher, catalog #: 12-545-80 CIR-1) in a 24-well plate were coated with 4 mL per plate or 500 μL per well of 50 μg/ml poly-D-lysine (PDL, Sigma, catalog #: P6407) at 4°C overnight. The next day PDL was removed and the plates were washed with sterile distilled water two times. Then they were coated with the same volumes of 5 μg/mL Laminin (Sigma, catalog #: L2020) overnight at 4°C. The plates were then put into a 37°C cell incubator while preparing the neurons for 1 h and Laminin was removed immediately before plating neurons.

Neurons were extracted according to instructions from BrainBits. Briefly, connective tissues of cortex were digested with 2 mL of 2 mg/mL of papain (Brain Bits, catalog #: PAP/HE-Ca) at 30°C water bath for 15 min, gently swirling every 5 min. Papain was then removed without disturbing the tissue at the bottom of the tube. The HEB media was then added to the tissue tube. The salinized Pasteur pipette was then used to triturate 30 times very slowly and carefully to not add air bubbles with the tip in the middle of the tube. The undispersed pieces were allowed to settle for one minute. The supernatant with dispersed cells were then transferred to a sterile 15 mL tube. This was spun down at 200 × g for one minute and the supernatant was discarded. Afterwards 1-2 mL of NbActiv1 was added while carefully avoiding air bubbles. Neurons were plated at 1 million per 10-cm dish or 25,000 per well of 24-well plate. The neuronal culture media contained the following: 100 mL Neurobasal A (Gibco, catalog #: 21103049), 2 mL B27 (Gibco, catalog #: 17504044), 0.25 mL GlutaMAX (Gibco, catalog #: 35050061) and 1 mL Penicillin-Streptomycin (Hyclone, catalog #: sv30010). Additionally, cytosine β-D-arabinofuranoside hydrochloride (Ara-C hydrochloride) (Sigma, catalog #: C6645) was added from day-in-vitro (DIV) 3 at a final concentration of 2 μM each time as half of the media was changed every three days. At DIV 6, lentivirus transduction was carried out to the neurons as indicated; the multiplicity-of-infection (MOI) was at 10, that is, the ratio of lentivirus count to neuron cells count in each well. At DIV 12, neurons were subjected to protein extraction for co-IP or immunofluorescence staining for confocal microscopy.

#### Mouse brain homogenization

C57BL/6J mice (Jackson laboratory) at 8–10 weeks were sacrificed, and the cortex was isolated and stored at −80°C. On the day of experiments, the cortex was thawed on ice and homogenized in homogenization buffer (25mM Tris-HCl pH 7.5, 150 mM NaCl, 1% NP-40, 0.5% sodium deoxycholate, 0.1% SDS supplemented with Roche complete protease inhibitor cocktail) using a plastic micro tissue homogenizer, and then sheared by passing 10 times through a 21G needle. Homogenates were centrifuged at 800 g for 10 min at 4°C and supernatants were collected. The pellet was re-homogenized in additional homogenization buffer and centrifuged at 800 × g for 10 min at 4°C, and the supernatant was collected again. The supernatants were combined and rotated at 4°C for 2 h, and then centrifuged at 18,000 × g for 30 min at 4°C. The resulting supernatant was collected as mouse brain homogenate, and its protein concentration was determined by a MicroBCA assay. The animal studies followed the guidelines of the Institutional Animal Care and Use Committees (IACUC) at Case Western Reserve University.

#### Confocal immunofluorescence

To label cell surface GABA_A_ receptors, primary neurons that were cultured on coverslips, were fixed with 2% paraformaldehyde in DPBS, blocked with goat serum for 0.5 h at room temperature, and labeled with 100 μL of appropriate anti-α1 (Synaptic Systems, catalog #: 224203), β2/3 (Millipore, catalog #: 05-474), or γ2 (Synaptic Systems, catalog #: 224003) antibodies (1:200) for 1 h without detergent permeabilization. Afterwards, they were incubated at room temperature with 500 μL (1:400) of Alexa 594-conjugated goat anti-rabbit antibody (ThermoFisher, catalog #: A11037), or Alexa 594-conjugated goat anti-mouse antibody (ThermoFisher, catalog #: A11032) for 1 h. Afterwards, cells were permeabilized with saponin (0.2%) for 5 min and incubated with DAPI (1 μg/mL) for 3 min to stain the nucleus. The coverslips were then mounted and sealed. For confocal immunofluorescence microscopy, an Olympus IX-81 Fluoview FV1000 confocal laser scanning system was used. A 60× objective was used to collect images using FV10-ASW software. Quantification of the fluorescence intensity was achieved using the ImageJ software from the NIH.

#### Biotinylation of cell surface proteins

To perform biotinylation assay, 6-cm dishes were coated in a 300-fold dilution of poly-L-lysine (Fisher, catalog #: ICN15017710) at 2 mL per plate for one hour at 37°C. Plates were rinsed twice with DPBS and allowed to air dry. The cells were plated on the coated dishes and incubated for 48 h post-transfection. The intact cells were washed one time with ice-cold DPBS and incubated with the membrane impermeable biotinylation reagent Sulfo-NHS SS-Biotin (0.5 mg/mL; APExBio, catalog #: A8005) in PBS containing 0.1 mM CaCl_2_ and 1 mM MgCl_2_ (PBS+ CM) for 30minat 4°C to label surface membrane proteins. In order to quench the reaction, the cells were incubated with 1.5 mL of 50 mM glycine in ice-cold DPBS-CM twice for 5 min at 4°C. They were then washed twice with DPBS. The sulfhydryl groups were then blocked by incubating the cells with 5 nM N-ethylmaleimide (NEM) in DPBS-CM for 15 min at room temperature and then the liquid was removed. Cells were scraped off plates and solubilized overnight at 4°C in lysis buffer (Tris–HCl, 50 mM; NaCl, 150 mM pH 7.5, 2 mM DDM) supplemented with Roche complete protease inhibitor cocktail and 5 mM NEM. The next day the lysates were centrifuged at 16,000 × g for 10 min at 4°C to pellet cellular debris. The supernatant was saved, and the protein concentration was measured with a MicroBCA assay. Biotinylated surface proteins were affinity-purified from the above supernatant by incubating for 2hat 4°C with 50 μL of immobilized neutravidin-conjugated agarose bead slurry (Fisher, catalog #: PI29200). The samples were then subjected to centrifugation (5,000 × g, 3 min). The beads were washed three times with buffer (Triton X-100, 1%; Tris–HCl, 50 mM; NaCl, 150 mM, pH 7.5) and three times further without Triton X-100. Surface proteins were eluted from beads by incubating for 30minat room temperature with 80 μL of LSB ⁄ Urea buffer (2x Laemmli sample buffer (LSB) with 100 mM DTT and 6 M Urea, pH 6.8) for SDS-PAGE and Western blotting analysis.

#### Cycloheximide-chase assay

Cycloheximide-chase assay to evaluate protein degradation in cells was carried out according to published procedures (Di et al., 2016). Briefly, cycloheximide (100 μg/mL), a potent protein synthesis inhibitor, was added to cell culture medium. HEK293T cells were then chased for the indicated time and harvested, and total proteins were extracted and subjected to SDS-PAGE and Western blot analysis.

#### Endoglycosidase H (endo H) enzyme digestion assay

To remove asparaginyl-*N*-acetyl-D-glucosamine in the N-linked glycans incorporated on the α1 subunit in the ER, total cell lysates were digested with Endo H enzyme (NEBiolab, catalog #: P0703L) with G5 reaction buffer at 37°C for 1h. The Peptide-N-Glycosidase F (PNGase F) enzyme (NEBiolab, catalog #: P0704L) treated samples served as a control for unglycosylated α1 subunits. Treated samples were then subjected to Western blot analysis.

#### Automated patch-clamping with IonFlux Mercury 16 instrument

Whole-cell currents were recorded 48 h post transfection of GT1-7 or HEK293T cells. Automated patch clamping was performed on the Ionflux Mercury 16 instrument (Fluxion Biosciences, California). The extracellular solution (ECS) contained the following: 142 mM NaCl, 8 mM KCl (Sigma, catalog #: P9541), 6 mM MgCl_2_ (Sigma, catalog #: M0250), 1 mM CaCl_2_ (Sigma, catalog #: C3306), 10 mM glucose (Sigma, catalog #: G8270), 10 mM HEPES (Sigma, catalog #: H3375). The intracellular solution (ICS) contained the following: 153 mM KCl (Sigma, catalog #: P9541), 1 mM MgCl_2_ (Sigma, catalog #: M0250), 5 mM EGTA (Sigma, catalog #: E3889), 10 mM HEPES (Sigma, catalog #: H3375). Briefly, cells were grown to 50 to 70 percent confluence on 10-cm dishes. Then 3 mL Accutase (Sigma Aldrich, catalog #: A6964-500mL) was added and the cells were incubated for 3 min at 37°C until the cells were floating as observed under microscope with minimal clumps. We then pelleted cells with centrifugation for 1 min at 200 × g, removed supernatant and resuspended cells in serum free medium HEK293 SFM II (Gibco, catalog #: 11686-029), supplemented with 25 mM HEPES (Gibco, catalog #: 15630-080) and 1% penicillin streptomycin (Hyclone, catalog #: sv30010). Cells were put on gentle shaking at room temperature for 0.5 to 1 h. Mercury 16 plates were prepared according to manufacture suggestions. Whole-cell GABA-induced currents were recorded at a holding potential of −60 mV, at 100 μM or 1 mM GABA concentration as indicated. The signals were acquired and analyzed by Fluxion Data Analyzer.

### Quantification and statistical analysis

All data are presented as mean ± SEM. Statistical significance was evaluated using Student’s *t*-test if two groups were compared and one-way ANOVA followed by post-hoc Tukey test if more than two groups were compared. A p< 0.05 was considered statistically significant. ∗, p< 0.05; ∗∗, p< 0.01.

## Data Availability

• All data reported in this paper will be shared by the lead contact on request. • This paper does not report original code. • Any additional information required to reanalyze the data reported in this paper is available from the lead contact on request.
